# Enhancing Safe Orthopedic Rehabilitation: Evaluating Documentation Practices for Mobilization and Weight-Bearing Protocols

**DOI:** 10.7759/cureus.98698

**Published:** 2025-12-08

**Authors:** Abdulshakor S Ali, Emmanuel O Oladeji, Gurvinder S Kainth

**Affiliations:** 1 Trauma and Orthopedics, Oxford University Hospitals NHS Foundation Trust, Oxford, GBR; 2 Trauma and Orthopedics, Surgery Interest Group of Africa, Lagos, NGA; 3 Trauma and Orthopedics, Tameside General Hospital, Ashton-under-Lyne, GBR

**Keywords:** boa guidelines, documentation standards, mobilization, musculoskeletal injuries, rehab protocol, weight bearing

## Abstract

Background

Accurate documentation of mobilization and weight-bearing status is critical for safe rehabilitation following orthopedic surgery or musculoskeletal injury. Poor or inconsistent recording can lead to miscommunication between teams, inappropriate rehabilitation prescriptions, and adverse patient outcomes. In August 2024, the British Orthopaedic Association (BOA) introduced updated standards to standardize terminology and enhance clarity across multidisciplinary teams. By auditing current practice against these BOA standards, this study identifies gaps in documentation and proposes targeted interventions to promote consistency, improve patient safety, and strengthen multidisciplinary care. This audit was conducted at a major trauma center in the UK.

Objectives

The objectives of this study are to evaluate compliance with BOA standards in documenting weight-bearing status and mobilization plans for orthopedic patients and to implement targeted educational interventions aimed at improving documentation practices, with their effectiveness assessed in a subsequent audit cycle.

Methods

A retrospective audit was conducted on 248 patients aged ≥16 years with operative or nonoperative orthopedic conditions affecting the pelvis, upper limbs, or lower limbs. Patients with spinal injuries or aged <16 years were excluded. Documentation was assessed against BOA criteria, including terminology use, clinical justification, quantification of limitations, duration of status, and rehabilitation protocols. Data were analyzed descriptively to determine compliance rates across documentation domains. Results are presented as proportions with 95% CIs.

Results

Of the 248 cases, only 95 (38.3%, 95% CI: 32.2-44.7) used standard BOA terminology: seven cases (3.0%, 95% CI: 1.2-6.0) documented “Unrestricted Weightbearing”, 30 cases (12%, 95% CI: 8.4-16.8) used “Limited Weightbearing” (LWB), and 58 cases (23%, 95% CI: 18.4-29.1) recorded “Non Weightbearing” (NWB). The remaining 153 cases (62%) used non-standard terms, with “Full Weightbearing” (FWB) being the most frequent at 104 cases (42%, 95% CI: 35.8-48.2). Out of the 248 cases audited, 119 patients (48%) required documentation to justify either NWB or LWB status. Among these, clinical justification was provided in 77 cases (65%), while 42 cases (35%) lacked appropriate documentation (95% CI: 57.0-72.5%). Quantification of LWB limitations was documented in 52 cases (68%), with 24 cases (32%) missing this detail (95% CI: 60.0-76.0%). From the total 248 cases, 118 patients required documentation specifying the duration of their NWB status. Of these, duration was recorded in 109 cases (92%), while nine cases (8%) lacked this information (95% CI: 87.0-96.5%). Additionally, among the 53 cases where documentation of walking aids and rehabilitation protocols was required, this information was recorded in 51 cases (96%) and absent in two cases (4%, 95% CI: 92.0-99.0%).

Conclusions

Initial compliance with BOA documentation standards was suboptimal, particularly regarding terminology and justification for restricted weight-bearing. Statistically significant gaps were identified across multiple domains. Targeted education and visual prompts may enhance documentation consistency and promote safer rehabilitation planning.

## Introduction

Clear instructions on post-injury mobilization and weight-bearing status are crucial in orthopedic care, as they directly affect rehabilitation, discharge planning, and patient safety. Noncompliance with immobilization instructions or premature weight-bearing can result in fracture fixation failure or instability in nonoperative treatment, leading to complicated fracture healing [1]. It may also increase the risk of falls, exacerbate injury severity, and negatively impact a patient’s rehabilitation journey. Conversely, delaying mobilization or unnecessarily prolonging immobilization can impede musculoskeletal recovery, affecting both short- and long-term outcomes, while increasing healthcare costs and placing additional pressure on healthcare services [2,3]. Therefore, a safe mobilization protocol tailored to the patient’s clinical context is essential.

Despite the recognized importance of accurately communicating weight-bearing instructions after musculoskeletal injury or orthopedic surgery, terminology describing weight-bearing status remains highly variable across institutions and practitioners [4]. The lack of standardized nomenclature contributes to miscommunication among healthcare professionals and confusion for patients, resulting in ambiguity in documentation and interpretation and inconsistency in clinical practice, which can compromise the safety and effectiveness of rehabilitation protocols.

In response to this variability, the British Orthopaedic Association (BOA) released updated standards in August 2024 to promote consistency in documentation practices. The guidelines provide consensus on documenting instructions, requiring clinical justification, quantification, functional and distance restrictions, duration, and details on walking aids or rehabilitation protocols to ensure patients receive clear, individualized guidance [5].

This clinical audit assesses adherence to BOA standards for recording mobilization and weight-bearing instructions in orthopedic patients and highlights the potential effectiveness of targeted educational interventions in improving documentation quality and supporting safer multidisciplinary rehabilitation practices.

## Materials and methods

A retrospective clinical audit was conducted over three months, from October to December 2024, at a Level 1 major trauma center. The audit aimed to assess adherence to BOA standards for recording mobilization and weight-bearing status among orthopedic inpatients and outpatients. Electronic patient records were reviewed to evaluate the presence, accuracy, and completeness of documentation related to mobilization plans and prescribed weight-bearing instructions.

Patients were eligible for inclusion if they were aged 16 years or older and had been assessed by the orthopedic team for operative or nonoperative conditions involving the pelvis, upper limb, or lower limb. Patients with spinal injuries or those under 16 years of age were excluded.

The audit was conducted in accordance with the BOA Standards for Mobilization and Weight-Bearing (August 2024). These standards recommend using standardized terminology, such as Non-Weightbearing (NWB), Limited Weightbearing (LWB), or Unrestricted Weightbearing (UWB), and require clinicians to provide justification when NWB or LWB restrictions are prescribed. The guidelines further advise that limitations be quantified by distance or functional capacity, that the intended duration and progression of weight-bearing status be documented, and that relevant walking aids and rehabilitation protocols be clearly recorded.

Data were analyzed descriptively to determine compliance rates across documentation domains. Results are presented as proportions with 95% CIs.

## Results

A total of 248 orthopedic patients were included in the audit, encompassing both inpatient and outpatient settings. The study cohort had a mean age of 64.9 ± 22.2 years (range: 17-99 years) and included 133 females (53.6%) and 115 males (46.4%). Most patients sustained lower limb fractures (178, 71.8%), followed by upper limb fractures (60, 24.2%) and pelvic fractures (10, 4%). Operative management was undertaken in 242 cases (98%), primarily involving surgical fixation, arthroplasty, or adjunctive procedures. The remaining six patients (2.4%) received conservative treatment for upper limb injuries. Documentation was most frequently completed by registrars.

An overview of compliance with the British Orthopaedic Association Standards for Trauma (BOAST) guidance regarding mobilization and weight-bearing after orthopedic surgery or musculoskeletal injury is presented in Table 1.

**Table 1:** Compliance pattern with BOAST guidance BOAST, British Orthopaedic Association Standards for Trauma

Domain	N	Compliant	N (%)	Noncompliant	N (%)
Nomenclature	248	95	38.30%	153	61.70%
Clinical justification	119	77	65%	42	35%
Quantification of limitations	76	52	68%	24	32%
Duration of limited weight-bearing	118	109	92%	9	8%
Documentation of walking aids and rehabilitation protocols	53	51	96%	2	4%

**Figure 1:**
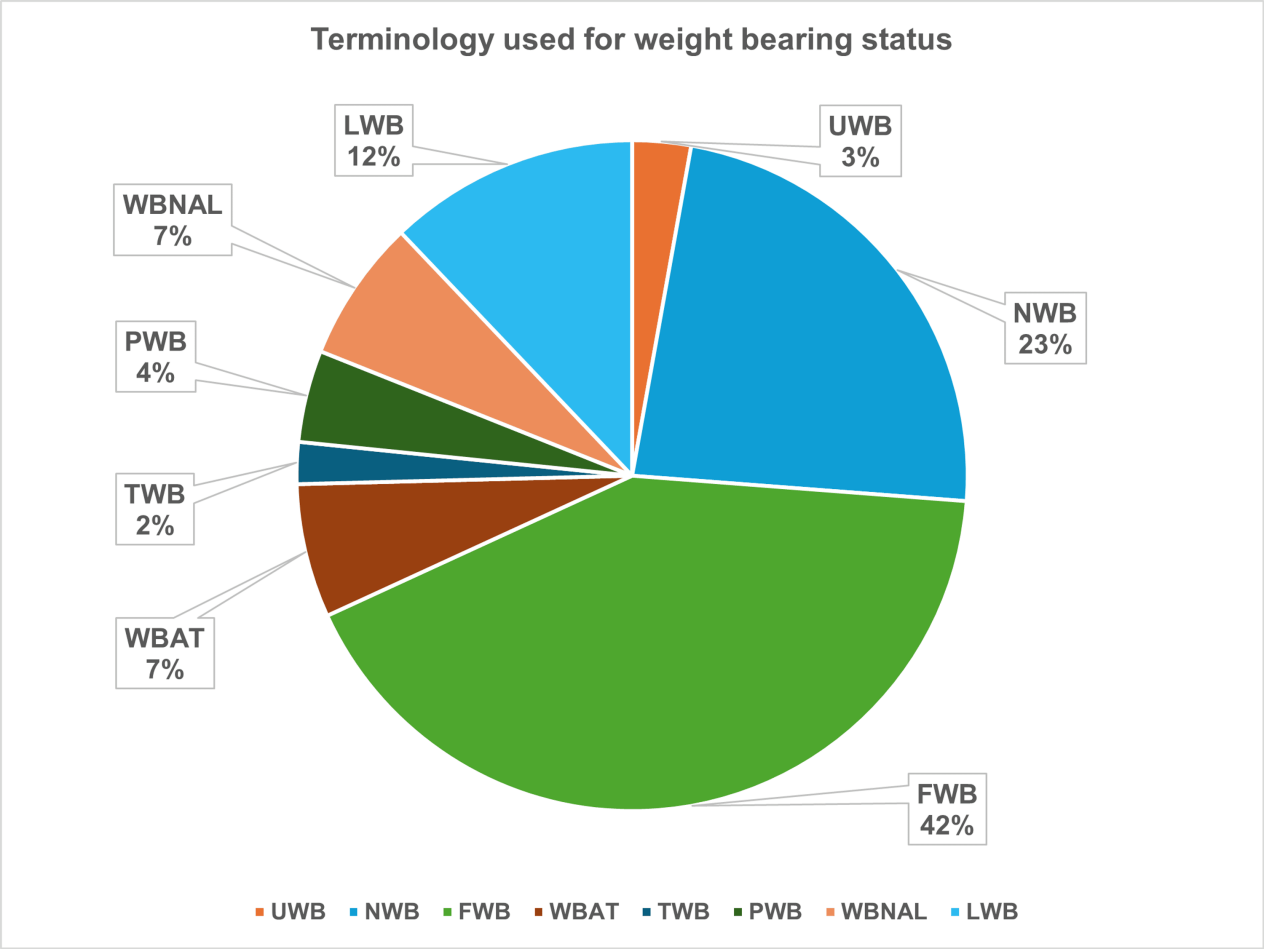
Distribution of terminology used for weight-bearing status in the operation notes FWB, full weight bearing; LWB, limited weight bearing; NWB, non-weight bearing; PWB, partial weight bearing; TWB, touch weight bearing; UWB, unrestricted weight bearing; WBNAL, weight bearing not allowed; WBAT, weight bearing as tolerated

In the analysis of terminology use across 248 cases, standard weight-bearing terminology was used in 95 cases (38%). This included seven cases (3%) documenting “Unrestricted Weightbearing” (UWB), 30 cases (12%) using “Limited Weightbearing” (LWB), and 58 cases (23%) recording “Non Weightbearing” (NWB). The remaining 153 cases (62%) used non-standard terms, with “Full Weightbearing” (FWB) being the most frequent at 104 cases (42%) (Figure 1).

Other non-standard terms recorded in 49 cases (20%) included partial weight-bearing (PWB), toe-touch weight-bearing (TTWB), weight-bearing not allowed (WBNAL), weight-bearing as tolerated (WBAT), and protected weight-bearing (PWB).

Of the 248 cases reviewed, clinical justification for NWB or LWB status was required in 119 cases; this was documented in 77 cases (65%), while 42 cases (35%) lacked justification. Among the 76 cases requiring quantification of LWB limitations, documentation was present in 52 cases (68%) and absent in 24 cases (32%).

Regarding the duration of NWB status, 118 cases were assessed. Documentation was available in 109 cases (92%), with only nine cases (8%) missing this detail. Among the 53 cases where documentation of walking aids and rehabilitation protocols was required, this information was recorded in 51 cases (96%) and was absent in two cases (4%).

**Figure 2:**
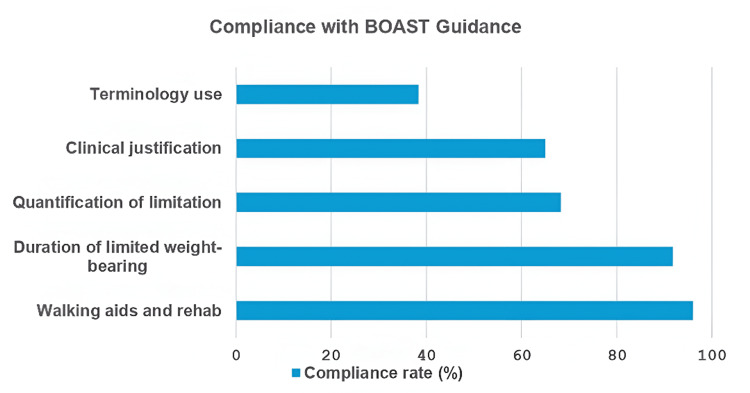
Compliance rate with BOAST guidance BOAST, British Orthopaedic Association Standards for Trauma

Overall, compliance with documentation standards varied across domains. Clinical justification for NWB/LWB status (65%) and quantification of LWB limitations (68%) were less consistently documented, whereas NWB duration (92%) and rehabilitation protocols (96%) demonstrated stronger compliance. These findings highlight both areas of good practice and opportunities for improvement in aligning with BOA standards (Figure 2).

Intervention

Following the initial audit and engagement with the multidisciplinary team to address identified documentation deficiencies, several targeted measures were implemented to improve compliance with established standards. Posters summarizing the BOA Standards for Mobilization and Weight-Bearing were displayed in key clinical areas, including the trauma meeting seminar room, outpatient fracture clinic, and theater computer room. Audit findings were presented to the clinical team to raise awareness of current practices and highlight areas for improvement.

Educational sessions were delivered to junior team members to reinforce the importance of accurate terminology and comprehensive documentation. Additionally, surgeons and trainees were encouraged to consistently use BOA-defined terms in operation notes and discharge summaries to ensure alignment with national guidelines and promote safe, effective patient rehabilitation. These interventions have been implemented; however, their effectiveness has not yet been measured. The impact will be formally assessed in the second audit cycle.

## Discussion

This audit demonstrated that documentation of mobilization and weight-bearing remains inconsistent compared to current standards. The most significant gaps were observed in adopting standardized terminology and in recording the rationale for, as well as the operational quantification of, restrictions. Only 95 cases (38.3%) used BOA-concordant terminology, with continued reliance on outdated phrases such as “full weightbearing,” which is no longer recommended. Clinical justification and quantification were documented in 77 (65.0%) and 52 (68%) of relevant cases, respectively, whereas duration limits and rehabilitation aids were recorded at very high rates (>90%). Overall, the pattern suggests that clinical teams consistently document the duration of weight-bearing restrictions and prescribed walking aids; however, explanations for the rationale behind weight-bearing limitations and guidance on daily functional expectations are less frequently recorded. These omissions are significant, as such information is essential to support patient understanding, engagement, and adherence to rehabilitation protocols.

These findings align with the purpose of the August 2024 BOA Standard on mobilization and weight-bearing, which aimed to remove ambiguous terms (e.g., Partial Weight Bearing (PWB), Toe-Touch Weight Bearing (TTWB), and Weight Bearing As Tolerated (WBAT)) in favor of three standardized terms, such as Non-Weightbearing (NWB), Limited Weightbearing (LWB), and Unrestricted Weightbearing (UWB), while also requiring documentation of (i) clinical justification; (ii) quantification of restriction; and (iii) duration, including planned progression [5]. The terminology is grounded in multi-stakeholder consensus formally recommending these prescriptions [4]. Our findings mirror the semantic drift observed in other early implementations, most notably the widespread use of “FWB” (Full Weight Bearing) when the clinical intention is actually “UWB” (Unrestricted Weight Bearing) [6]. This inconsistency underscores the need for standardized vocabulary in note templates and discharge summaries, which offers a simple yet effective early intervention to improve clarity and compliance.

The moderate rates of justification and quantification warrant focused attention because they directly relate to safety and patients’ ability to follow instructions. Multiple studies show that patients, especially older adults, struggle to achieve prescribed partial or limited loading without targeted instruction or feedback [7,8]; even motivated patients tend to under- or overload when guidance is expressed as percentages or kilograms. When simple, functional instructions are used (e.g., “indoors only,” “bed-to-chair transfers,” “no stairs”), adherence improves, and therapy teams can implement plans more consistently [5,9,10]. The service already demonstrates strong consistency in documenting duration and prescribed aids, suggesting that applying the same structured approach to include the rationale (“why”) and specific daily guidance (“what exactly”) is both practical and achievable.

Implementation science in orthopedics and other surgical specialties supports practical tools such as visual prompts and brief education and highlights additional high-impact changes. Using templated proformas (preferably electronic) and procedure-specific operation notes consistently enhances completeness and adherence to documentation standards across subspecialties [11-14]. Translating BOAST requirements into mandatory fields, such as drop-downs limited to NWB/LWB/UWB, structured prompts for justification, functional or distance limits, duration, and aids, should help reduce non-standard entries and prevent omissions. Aligning the periodic inpatient review schedule with BOAST creates checkpoints to reassess restrictions and prevent unnecessary physiotherapy delays [5]. Providing patients with discharge summaries that include mobilization instructions offers a clear and accessible reminder to support recovery.

Limitations

This audit has several limitations. Although nonoperatively managed patients were initially included, they were later excluded due to the logistical challenges of retrieving mobilization and weight-bearing information. Documentation for this group was often inconsistent and required extensive manual review of ward round entries and clinic letters, which limited data availability and reliability. As a result, the final cohort was predominantly composed of operatively managed patients, leading to underrepresentation of documentation practices in those treated nonoperatively.

Furthermore, mobilization and weight-bearing status were not clearly documented for 12 patients who underwent combined OrthoPlastics interventions for complex fractures with compromised soft tissues, as definitive fixation and grafting were still pending at that stage.

Despite these limitations, operation notes and discharge summaries form the basis of rehabilitation prescriptions in most pathways, and improving their quality and consistency remains clinically significant. This baseline reflects national trends observed during early BOAST adoption: while documentation of duration and prescribed aids is generally robust, terminology and the “why/how” fields remain inconsistent.

Addressing these gaps by eliminating non-standard terms, enforcing BOAST-compliant fields with functional or distance-based quantification, embedding automated review prompts, and sustaining targeted education initiatives is likely to yield measurable improvements on reaudit. Future cycles should also incorporate patient-centered outcomes, such as time to first mobilization, therapy delays due to unclear orders, falls, and length of stay, to assess whether improvements in documentation translate into safer and more efficient rehabilitation.

## Conclusions

Initial compliance with BOA standards for mobilization and weight-bearing documentation was suboptimal, with notable gaps in terminology use, clinical justification, and quantification. Targeted educational interventions and visual prompts have been introduced, and their impact will be evaluated in a planned reaudit. While this baseline audit highlights areas of inconsistency, it also demonstrates that documentation of duration and prescribed aids is generally robust. Standardized documentation remains essential for safe and effective rehabilitation, and embedding BOA-compliant practices into routine orthopedic workflows is likely to improve clarity and enhance patient safety.
